# Intronic *PAH* gene mutations cause a splicing defect by a novel mechanism involving U1snRNP binding downstream of the 5’ splice site

**DOI:** 10.1371/journal.pgen.1007360

**Published:** 2018-04-23

**Authors:** Ainhoa Martínez-Pizarro, Maja Dembic, Belén Pérez, Brage S. Andresen, Lourdes R. Desviat

**Affiliations:** 1 Centro de Biología Molecular Severo Ochoa UAM-CSIC, CEDEM, CIBERER, IdiPaz, Universidad Autónoma, Madrid, Spain; 2 Department of Biochemistry and Molecular Biology and the Villum Center for Bioanalytical Sciences, University of Southern Denmark, Odense, Denmark; The Chicago Medical School, UNITED STATES

## Abstract

Phenylketonuria (PKU), one of the most common inherited diseases of amino acid metabolism, is caused by mutations in the phenylalanine hydroxylase (*PAH*) gene. Recently, *PAH* exon 11 was identified as a vulnerable exon due to a weak 3’ splice site, with different exonic mutations affecting exon 11 splicing through disruption of exonic splicing regulatory elements. In this study, we report a novel intron 11 regulatory element, which is involved in exon 11 splicing, as revealed by the investigated pathogenic effect of variants c.1199+17G>A and c.1199+20G>C, identified in PKU patients. Both mutations cause exon 11 skipping in a minigene system. RNA binding assays indicate that binding of U1snRNP70 to this intronic region is disrupted, concomitant with a slightly increased binding of inhibitors hnRNPA1/2. We have investigated the effect of deletions and point mutations, as well as overexpression of adapted U1snRNA to show that this splicing regulatory motif is important for regulation of correct splicing at the natural 5’ splice site. The results indicate that U1snRNP binding downstream of the natural 5’ splice site determines efficient exon 11 splicing, thus providing a basis for development of therapeutic strategies to correct *PAH* exon 11 splicing mutations. In this work, we expand the functional effects of non-canonical intronic U1 snRNP binding by showing that it may enhance exon definition and that, consequently, intronic mutations may cause exon skipping by a novel mechanism, where they disrupt stimulatory U1 snRNP binding close to the 5’ splice site. Notably, our results provide further understanding of the reported therapeutic effect of exon specific U1 snRNA for splicing mutations in disease.

## Author summary

Splicing defects constitute a major cause of human disease. Mutations affecting conserved splicing sequences at exon-intron junctions are easily recognized as possibly pathogenic, whereas variants in exonic or intronic regions are difficult to classify without functional evidence provided by transcript analysis or in vitro analysis using minigenes. In this work, we sought out to study the pathogenicity of two novel intronic *PAH* variants identified in phenylketonuria patients. Both mutations resulted in exon skipping in minigenes. We demonstrate that U1snRNP70 binds to the intronic region and that this binding is abolished in the mutant sequences. Correction of the splicing defect was achieved using modified U1 snRNA perfectly complementary to each of the mutant sequences. The results extend the repertoire of natural U1 snRNP cellular functions by including its role as splicing enhancer via binding downstream of the natural 5’ splice site. In addition, our results correlate with the described therapeutic effect of modified U1snRNP for splicing mutations in different genes, thus having a significant impact in the development of specific therapies for splicing defects.

## Introduction

The significant contribution of splicing defects to human disease is to date well established. Pathogenic splicing mutations include both genomic variants located in consensus splicing sequences (5’ splice site, 3’ splice site, branch point, and polypyrimidine tract), as well as other variants, located in exonic or intronic regulatory splicing elements, that modulate spliceosome recruitment [1–3]. These cis-regulatory elements are referred to as exonic or intronic splicing enhancers or silencers (ESE, ISE, ESS, or ISS). These elements are recognized by trans-acting factors including the serine/arginine-rich domain-containing (SR) protein and heterogeneous nuclear ribonucleoprotein (hnRNP) families, which usually act in concert and may display cooperative or antagonistic effects during spliceosome assembly. Together they define splice site selection and alternative splicing decisions [4]. In constitutively spliced exons, auxiliary trans-acting splicing factors are required when the conserved splicing signals are weak, i.e. when the 5’ or 3’ splice sites sequences deviate from the consensus altering the splice site strength.

At present, there is ample evidence of apparently neutral or silent variants, or even predicted missense mutations, that in fact cause disease by altering enhancer or silencer regions, thus affecting the splicing process. In these cases, the so-called splicing code overrules the genetic code that predicts an amino acid substitution [4]. There are several ways by which a point mutation in exonic or intronic regions can cause aberrant splicing, including creation or activation of alternative splice sites, weakening of canonical splice sites promoting the use of a natural cryptic splice site, or activating the inclusion of intronic pseudoexons which are normally not included in the mature mRNA [1].

Most of the reported disease-causing splicing mutations affect the 5’ splice donor site [5], hindering correct initiation of spliceosome formation, that occurs via recognition of this site by the U1 snRNP (small ribonucleoprotein particle). U1 snRNP is composed of a 164 bp long U1 snRNA and several proteins, namely U1-A, U1-70K, and U1-C as well as Smith antigen (Sm) proteins [6]. The 5’ end of U1 snRNA binds by complementarity to the conserved 5’ splice site, spanning the last 3 nucleotides of the exon, and nucleotides +1 to +6 of the intron. There is probably a minimal number of 5–6 base pairing to U1snRNA for a functional 5’ splice site, but the different nucleotide positions are not functionally equivalent or equally conserved, and they appear also to be interdependent [7, 8]. Mutations that lower the complementarity to U1 snRNA usually cause splicing defects, and even mutations to nucleotides such as +3A>G, where +3G is present in approximately half of functional splice sites, may cause complete inactivation [8, 9]. Recently, U1 cellular functions have extended beyond its involvement in the splicing process, as it was shown that it protects transcripts from premature cleavage and polyadenylation and it can also promote transcription [10].

The implementation of next generation sequencing technologies in clinical diagnostics has revealed the difficulty in ascribing pathogenicity to novel variants, especially in intronic regions, which are known as sequence variants of unknown significance (VUS). Usually, a combination of *in silico* tools, mostly focused on protein features for coding variants, or in the alteration of the conserved 3’ or 5’ splice sites, is used to distinguish pathogenic variants. However, correct prediction of a potential effect on splicing of variants located in non-canonical splicing regulatory elements is elusive. Recently, using a machine-learning approach, a computational model was developed to predict the impact on splicing of any intronic or exonic variant, taking into account features in the exons and neighbouring introns, which often influence exon inclusion [11]. Even so, functional assays commonly performed using minigenes are still mandatory to confirm a splicing defect, when transcript analysis in patient samples is not possible [3, 12]. In addition, the exact consequences of a splicing mutation at the transcript level (exon skipping, activation of alternative splice sites, pseudoexon inclusion, intron retention, etc.) are mostly unpredictable. In this respect, minigenes are also relevant tools for the analysis of the pathogenic mechanism, confirming the role of *cis* and *trans-*acting factors in splicing regulation and providing a rationale for the implementation of specific therapies.

Among the main therapeutic strategies based upon splicing modulation is the use of splice-switching antisense oligonucleotides (SSOs) [13] and of adapted U1 snRNA [14, 15]. Both target the pre-mRNA aiming to influence the ratio between mRNA isoforms to restore normal splicing or to favour potentially therapeutic variants. SSOs are designed to base-pair with specific splice sites or splicing regulatory sequences to hinder their recognition by the spliceosome. Clinical trials using different chemistries have produced encouraging results for Duchenne muscular dystrophy and spinal muscular atrophy, with two SSOs recently approved by the FDA, indicating that these approaches should be applicable to additional mis-splicing defects [16].

In the past few years, evidence has accumulated supporting the use of U1 snRNAs with a modified 5’ tail, that base-pairs exactly with mutant donor 5’ splice sites, as a strategy to effectively correct splicing defects of 5’ splice site mutations [14, 15]. However, this correction is mutation specific and the adapted U1 snRNA can potentially bind to other 5’ splice sites, thus altering other splicing events. Subsequently, Pagani and co-workers generated exon specific U1 snRNAs (ExSpeU1) with engineered 5’ tails binding at non-conserved intronic sequences downstream of the exon, which were able to correct different exon-skipping mutations located at exonic or intronic sites [17, 18]. In this case, U1snRNA binding mediates 5’ splice site activation thus favouring exon and intron definition. In vivo, ExSpeU1s are assembled as U1-like particles and their splicing rescue activity is dependent on the U1 snRNP 70 (U1-70K) protein and on the loop structure of the U1 snRNA [18], but the exact mechanism remains unclear.

Phenylketonuria, one of the most common inherited diseases of amino acid metabolism, is caused by a defect in the phenylalanine hydroxylase (*PAH*) gene, and approximately 13% of the mutations affect conserved 3’ and 5’ splice sites, and are thus recognized as causing splicing defects (HGMD Professional Release 2017.1). In addition, some studies have revealed that synonymous or missense mutations may cause a splicing defect [19–21]. *PAH* exon 11 was recently identified as a vulnerable exon due to a weak 3’ splice site implying that different exonic mutations affected exon 11 splicing by altering splicing regulatory elements distributed throughout the exon [22].

In this work, we have identified a splicing regulatory element in intron 11, which ultimately determines exon 11 recognition and mediates the disease-causing effect of the intronic variants c.1199+17G>A and c.1199+20G>C identified in PKU patients with no obvious pathogenic effect a priori. We show that this element functions by recruiting U1 snRNP to stimulate recognition of the upstream splice site.

## Results

### Mutation identification and in silico predictions

The c.1199+17G>A variant has previously been described [23] and was detected in 3 compound heterozygous hyperphenylalaninemia patients referred to the diagnostic laboratory in Madrid. The c.1199+20G>C variant is located in the same region as a private mutation identified in a patient from USA [24]. Both variants are reported in dbSNP with no associated MAF or indication of clinical significance. None of them are present in ExAc.

The possible pathogenic effect of the two intronic variants on splicing was examined using Alamut software, ESEfinder, and HSF program. The c.1199+17G>A variant is predicted to disrupt binding motifs for SRSF1 and SRSF7 splicing factors, create a Tra-2β binding site, and abolish hnRNPA1 binding sites while creating a novel one. For c.1199+20G>C, disruption of a SRSF7 binding site is predicted (Table 1).

**Table 1:** Splicing factors binding sites in the *PAH* intronic region surrounding the c.1199+17G>A and c.1199+20G>C variants, predicted with ESEFinder (http://rulai.cshl.edu) and HSF (http://www.umd.be/HSF3/) programs. Effects of variants and deletions introduced in minigenes. WT; wild type.

	SRSF1	SRSF1 (IgM-BRCA1)	SRSF2	SRSF5		SRSF7		Tra-2β	hnRNP A1		
**WT**	+	+	+	+	+	+	+	-	+	+	-
**+17G>A**	-	-	+	+	+	+	-	+	-	-	+
**+20G>C**	+	+	+	+	+	+	-	-	+	+	-
**+13del7**	-	-	+	+	+	-	-	-	-	-	+
**+17del6**	-	↓	+	+	-	+	-	-	-	-	-
**+20del5**	+	+	-	+	↓	+	-	-	+	+	-

A plus (+) sign denotes the predicted presence and a minus (-) sign indicates absence or loss of a binding site for the splicing factor. Downward arrows indicate a decrease in the predicted binding strength of the splicing factor.

### Minigene analysis

**Fig 1:**
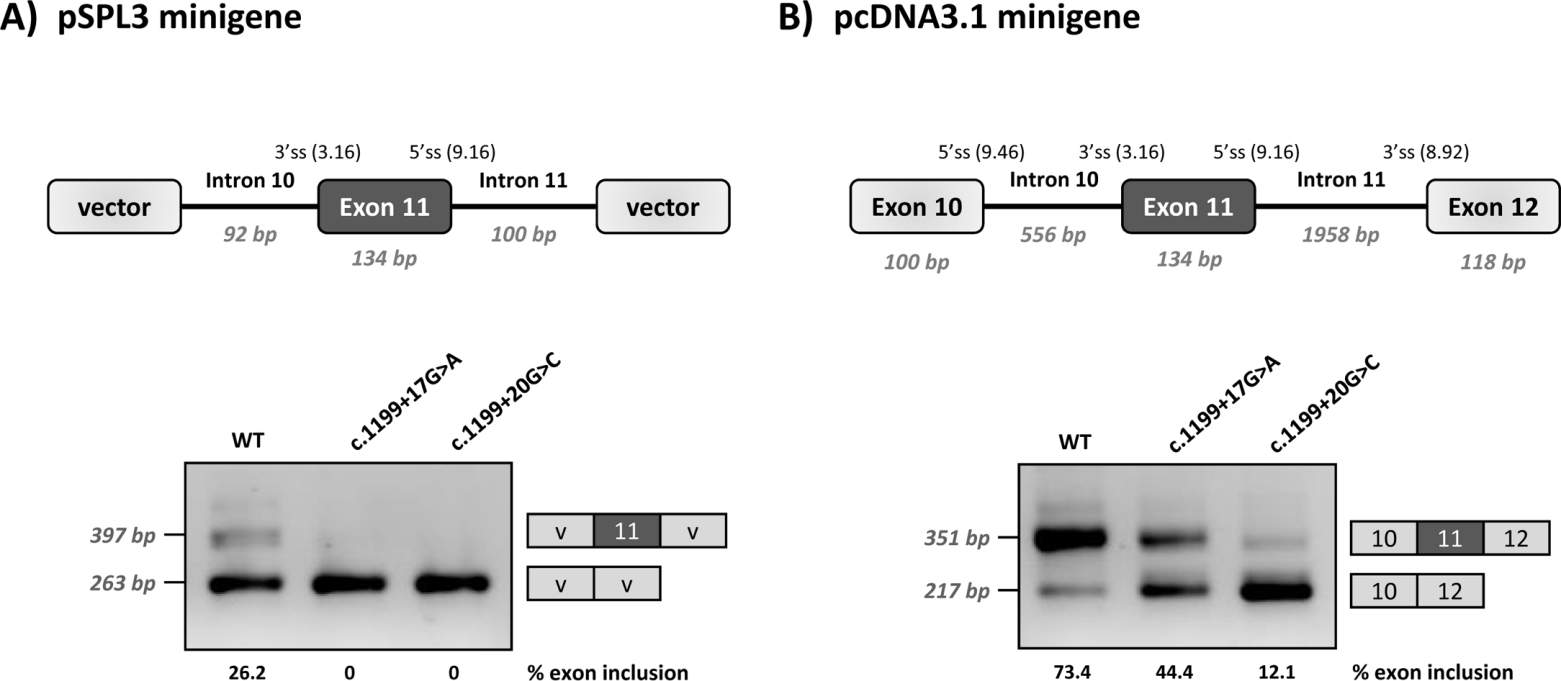
Minigene analysis of the c.1199+17G>C and c.1199+20G>C variants. Panel A shows the schematics of the pPSL3 construct and the results after transfection in Hep3B cells of wild-type (wt) and mutant minigenes. Panel B shows the schematics of the pcDNA3.1 construct and the results in Hep3B cells. The splice scores according to MaxEnt program (http://genes.mit.edu/burgelab/maxent/Xmaxentscan_scoreseq.html) are indicated for each splice site. On the right of each gel is the schematic drawing showing the identity of the bands confirmed by sequencing analysis The estimated percentage of exon inclusion is shown below each lane. V, vector sequences.

Functional analysis of c.1199+17G>A and c.1199+20G>C was performed using two different minigene constructs. As shown in Fig 1, the +17A and +20C variants result in variable degrees of *PAH* exon 11 skipping, confirming that their pathogenic nature is caused by a splicing defect. In the wild type minigenes, residual exon 11 skipping is observed. This is due to a naturally weak 3’ splice site, as previously described [22]. Moreover, the exon 11 5’ splice site is also not optimal, with suboptimal nucleotides at positions +3 and +6. The weakness/vulnerability of exon 11 is particularly well reflected in the low inclusion rate of wild type exon 11 in the pSPL3 minigene that carries a shorter *PAH* genomic sequence and does not harbour the natural flanking splice sites. The wild type pcDNA3.1 minigene that has a more normal *PAH* structure as it includes the flanking exons, also displays low levels of exon 11 skipping.

**Fig 2:**
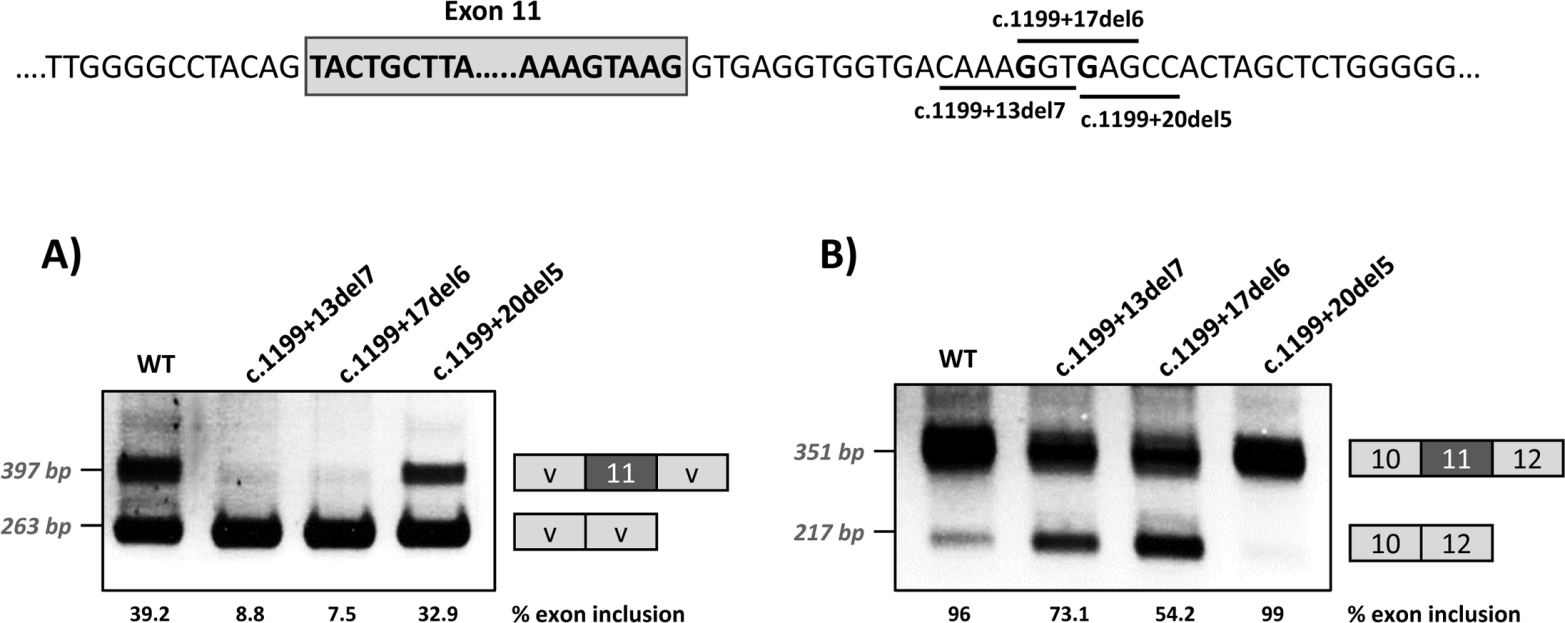
Effect of intronic deletions on minigenes splicing profile. Deletions c.1199+13del7, c.1199+17del6 and c.1199+20del5, shown in the scheme above, were introduced in the pSPL3 (A) or pcDNA3.1 (B) wild-type minigenes and the effect on splicing examined after transfection in Hep3B cells. The estimated percentage of exon inclusion is shown below each lane. On the right of each gel is the schematic drawing showing the identity of the bands. V, vector sequences.

With the aim of investigating the mechanism underlying the exon skipping defect, we performed targeted mutagenesis in the minigenes. First, we performed deletion mutagenesis in the intronic region of the wild-type minigenes, eliminating nucleotides +13 to +19 (c.1199+13del7), nucleotides +17 to +22 (c.1199+17del6) or nucleotides +20 to +24 (c.1199+20del5) in order to reveal potential splicing regulatory elements in this region. The disruption of the predicted splicing factor binding sites for each deletion mutant is shown in Table 1. Both the c.1199+13del7 (deletion of nucleotides +13 to +19) and the c.1199+17del6 (deletion of nucleotides +17 to +22) had a deleterious effect on exon inclusion, mimicking the effect of the point mutations c.1199+17G>A and c.1199+20G>C, while the deletion of nucleotides +20 to +24 (c.1199+20del5) had no detectable effect (Fig 2). The results indicate that the intronic nucleotides +13 to +20 form part of a regulatory region required for correct exon 11 recognition.

**Fig 3:**
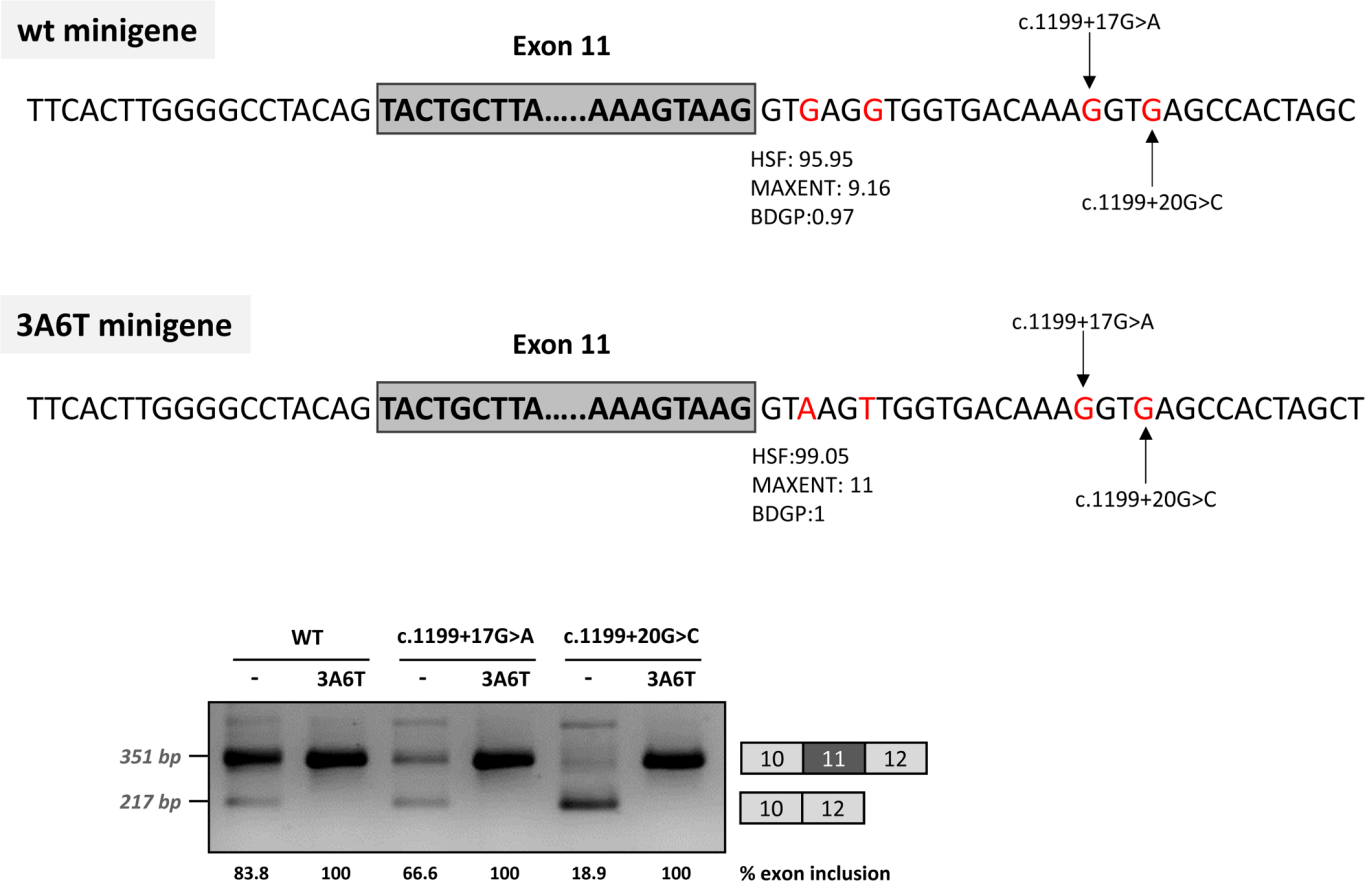
Effect of the optimization of the 5’ splice site of exon 11 on minigenes splicing profile. The splicing score of exon 11 5’ splice site was optimized in the 3A6T minigene by introducing the c.1199+3G>A and c.1199+6A>T changes as shown in the above panel, along with the predicted scores calculated with HSF (http://www.umd.be/HSF3/), MaxEntScan (http://genes.mit.edu/burgelab/maxent/Xmaxentscan_scoreseq.html) and BDGP (http://www.fruitfly.org/seq_tools/splice.html). The gel shows the RT-PCR results after transfection of the wild type (wt) and mutant pcDNA3.1 minigenes with and without the optimized 5’ splice site. The estimated percentage of exon inclusion is shown below each lane. On the right of the gel is the schematic drawing showing the identity of the bands.

In addition, because we speculated that the effect of the two mutations could be dependent on the suboptimal nature of the natural 5’ splice site, we optimized the 5’ splice site strength by replacing the suboptimal nucleotides at the +3 and +6 position in the pcDNA3.1 minigenes, to investigate if this could counteract the splicing defect caused by the intronic variants. The guanosine at c.1199+3 was replaced by adenine and the guanosine at c.1199+6 was replaced by thymine. These substitutions increased the maximum entropy score of the natural *PAH* exon 11 5’ splice site from 9.16 to 11 (Fig 3). In the +17 and +20 mutant minigenes, 100% exon inclusion was observed with these substitutions. Moreover, in the wild-type minigene the substitutions abolished the residual exon skipping completely (Fig 3).

Taken together, these data suggest that the c.1199+17G>A and c.1199+20G>C mutations disrupt the binding of a splicing factor which is required for correct recruitment of the spliceosome to the suboptimal *PAH* exon 11 5’ splice site. This can be compensated by increasing the strength of the 5’ splice site (i.e. by increasing the binding affinity for U1 and other snRNPs, which are recruited to the 5’ splice site during the splicing process).

### RNA affinity studies

**Fig 4:**
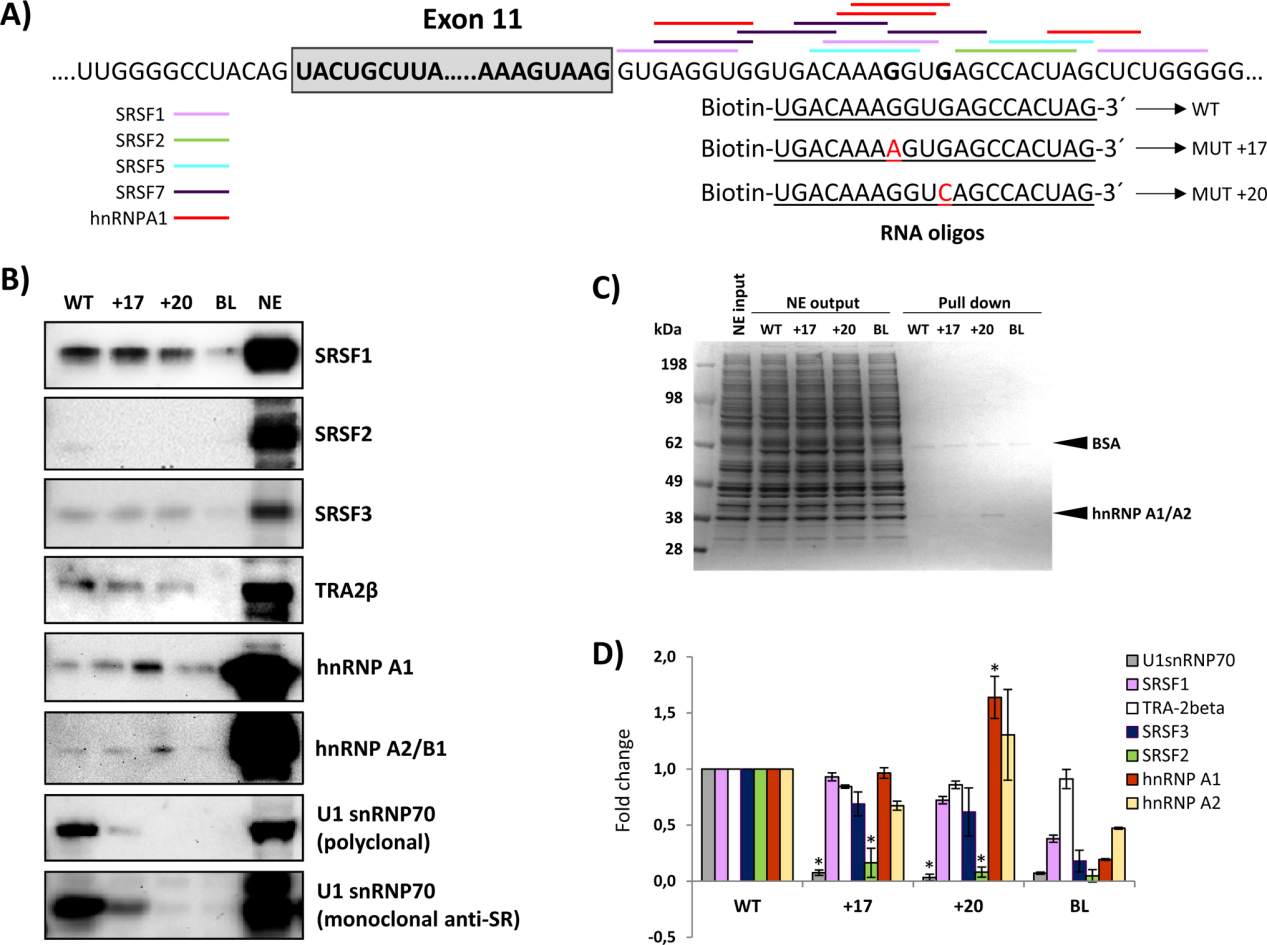
RNA oligonucleotide affinity studies. A) Schematic representation of the exon 11-intron 11 junction, the predicted binding sites for splicing factors and the RNA oligonucleotides used; B) Western blot gels after pull-down experiments; the blots shown are representative results from three independent pull-down experiments; C) Coomassie stained gels; 15 μg of HeLa nuclear extract (NE input), corresponding to 1/50 of the total nuclear extract used as input per pull-down reaction, equal amounts of nuclear extract collected after the binding reaction (NE output), and 7.5 μl (1/6) of the eluates were loaded and separated on an SDS-PAGE gel, and stained with Coomassie; D) Quantification of the pull down experiments: the intensity of the signal from western blots was quantified and normalized to the signal obtained from the pull-down reaction with the WT sequence. Student t-test was used to evaluate the differences, * p<0.05. BL and NE indicate control lanes without RNA oligonucleotides or with nuclear extract alone, respectively.

To identify the splicing factor(s) that may bind to the region where both intronic mutations are located, we performed RNA oligonucleotide binding studies. RNA oligonucleotides containing the wild type or the mutated c.1199+17A or c.1199+20C sequences were incubated in HeLa cell nuclear extract. After elution, proteins bound to each oligonucleotide were analysed by SDS-PAGE and Western blotting. We tested the presence of SRSF1, SRSF2, SRSF3, SRSF5, SRSF7, U1snRNP70, Tra-2β, hnRNPA2/B1, hnRNPI, hnRNPL, hnRNPH, hnRNPE2 and hnRNPA1. The results showed binding of SRSF1, SRSF3 and Tra-2β, with no significant differences between wild type and mutant sequences (Fig 4). SRSF2 exhibits very weak binding to the wild type sequence, which is almost undetectable for the mutant sequences. hnRNPA1 showed increased binding to the c.1199+20c mutant sequence. Interestingly, the analysis revealed strong binding of U1-70K to the wild type sequence, which was abolished by both mutant sequences (partly for c.1199+17a and completely for c.1199+20c) (Fig 4). This result was reproduced using two different antibodies, a polyclonal anti- U1-70K and an anti-SR monoclonal antibody.

**Fig 5:**
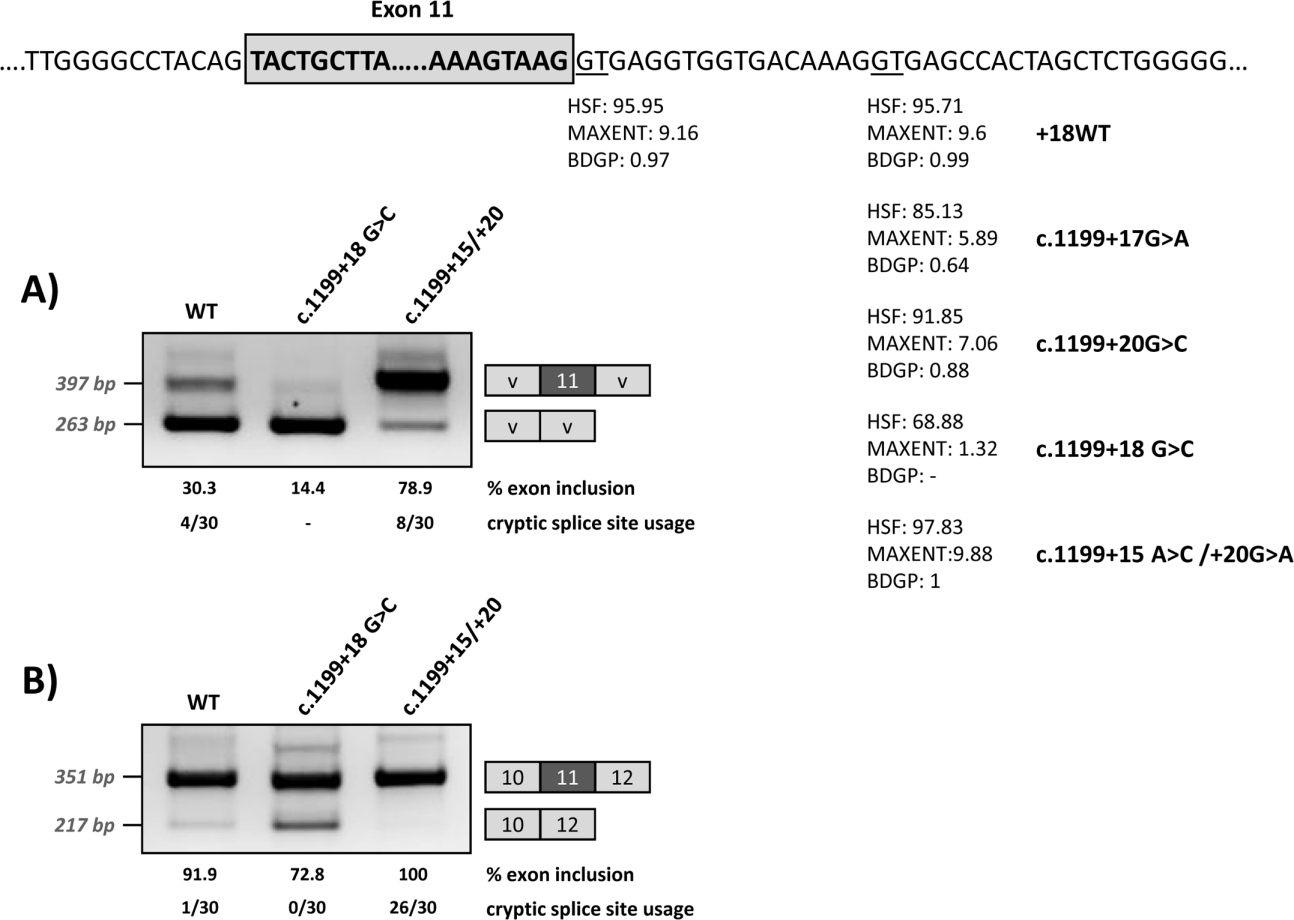
Effect of the modification of the intronic cryptic splice site on minigene splicing profile. The upper panel shows the location and predicted splice scores of the natural and cryptic (wild type and with the different mutations) splice sites. The intronic cryptic splice site was either abolished by elimination of the GT (c.1199+18G>C mutation) or optimized (c.1199+15A>C/+20G>A mutations). The gels show the RT-PCR results after transfection of the wild-type and modified pSPL3 (A) or pcDNA3.1 (B) minigenes. On the right of the gel is the schematic drawing showing the identity of the bands. HSF: Human Splice Finder (http://www.umd.be/HSF3/HSF.html); MAXENT: MaxEntScan (http://genes.mit.edu/burgelab/maxent/Xmaxentscan_scoreseq.html); BDGP: Berkeley Drosophila Genome Project (http://www.fruitfly.org/seq_tools/splice.html). The estimated percentage of exon inclusion and the cryptic splice site usage (number of clones in which splicing occurred at the +18 splice site out of total analysed, after subcloning and sequencing the exon inclusion amplified product) are shown below each lane.

Closer inspection of the intronic sequence revealed a potential binding site for U1 snRNA surrounding the GT nucleotides at positions +18 and +19, corresponding to a high 5’ splice site score in all prediction programs (Fig 5). Both point mutations create mismatches to the U1 snRNA consensus motif, decreasing the predicted splice site score.

### Cryptic splice site modification

In order to determine whether binding of U1 at the cryptic splice site at +18 is indeed relevant for exon 11 recognition, we modified by mutagenesis this region in the wild type minigenes, either abolishing the GT (c.1199+18G>C mutation) or strengthening the splice site score (c.1199+15A>C/+20G>A) (Fig 5). After transfection, RT-PCR analysis showed that disruption of the U1 snRNA binding motif results in increased exon skipping, while increasing the strength of the U1 snRNA motif favours exon inclusion (Fig 5).

To investigate the possible use of the intronic U1 binding site as a cryptic splice site, we cloned the PCR band corresponding in size to exon 11 inclusion for the wild type and mutant minigenes shown in Fig 5. Sequencing analysis of the PCR bands obtained for wild type minigenes identified 4/30 (pSPL3 minigene) and 1/30 clones (pcDNA3.1 minigene) in which splicing had indeed occurred at the cryptic splice site +18. For the c.1199+18G>C mutant minigene, in which the cryptic splice site is abolished, all the clones analysed showed splicing at the natural 5’ splice site, as expected. In the case of the c.1199+15A>C/+20G>A mutant minigene, cloning and sequencing analysis identified 8/30 (pSPL3 minigene) and 26/30 (pcDNA3.1 minigene) clones in which the modified cryptic splice site with an optimal 5’ splice score is used instead of the natural splice site. Sequence analysis of the PCR products obtained from the pcDNA3.1 minigenes also identified some clones with an additional PCR product corresponding to the inclusion of a 25 bp intronic region (corresponding to nucleotides c.1199+538_+562), which could be a minigene-derived artefact or a cryptic exon. This transcript was identified in 5/30 and 6/32 clones resulting from the wild type and c.1199+18G>C minigenes, respectively. It was not detected for the c.1199+15A>C/+20G>A mutant minigene.

Thus, in spite of its high splice site score and the ability to bind U1snRNP indicated by the RNA-affinity studies described above, in minigenes the intronic U1 binding site is used at a very low frequency as a cryptic splice site in the wild type sequence context. We investigated the situation *in vivo* analysing the endogenous *PAH* transcripts in a human liver sample and in hepatoma cell lines Hep3B and HepG2, the latter treated or not with cycloheximide to block nonsense-mediated decay. In all cases, analysis by capillary gel electrophoresis and/or sequencing after subcloning of the amplified transcript showed that only the natural 5’ splice site is used. Some residual exon 11 skipping was also observed in liver and in hepatoma samples, as previously described [25]. In the liver sample we also detected the intronic 25 bp insertion, thus it appears to be a natural cryptic exon.

Overall, the results confirmed that binding of U1 at the cryptic site is necessary for efficient exon 11 recognition and they indicated that the U1 binding site is not or marginally used as a cryptic splice site in a wild-type context both *in vitro* and *in vivo*.

**Fig 6:**
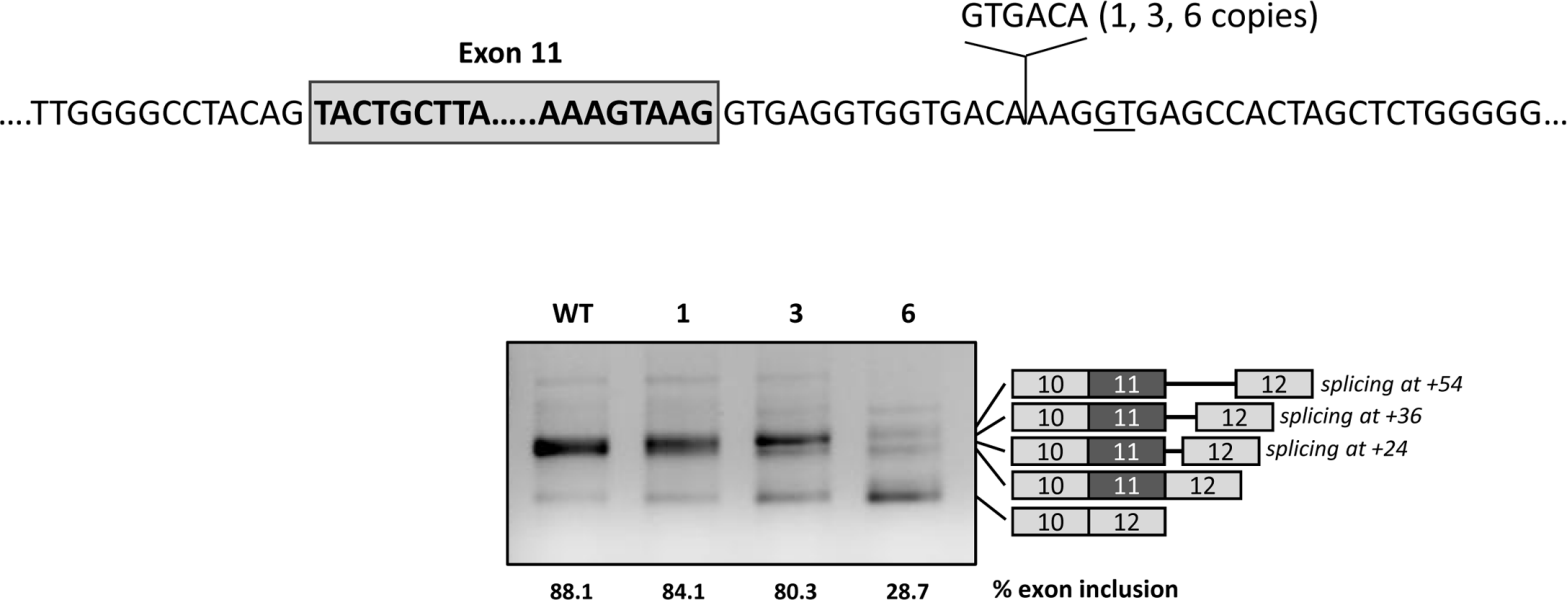
Effect of distance of the intronic regulatory region to the natural 5’ splice site. The upper panel shows the location and sequence of the spacer introduced in the pcDNA3.1 wild type minigene between the natural and the cryptic splice sites (GT underlined). The gel shows the results after transfection of the wild-type (WT) minigene and the constructs with 1, 3 or 6 spacers in Hep3B cells. On the right of the gel is the schematic drawing showing the identity of the bands verified by sequence analysis, that showed usage of both the natural and the cryptic splice site (at +24 with 1 spacer, at +36 with 3 spacers and at +54 with 6 spacers). The estimated percentage of exon inclusion is shown below each lane.

The functionality of the U1-mediated intronic splicing enhancer region most probably depends on the distance to exon 11. To investigate this, we tested the effect of expanding this distance by inserting 1, 3 and 6 copies of a 6 bp sequence upstream of the U1 binding site (Fig 6). The results show increased exon 11 skipping with increasing number of copies of the spacer. With all three constructs (1, 3 and 6 copies of the spacer) we could detect transcripts corresponding to usage of the cryptic splice site (now located at +24, +36 and +54, respectively).

### Overexpression of adapted U1 snRNA

**Fig 7:**
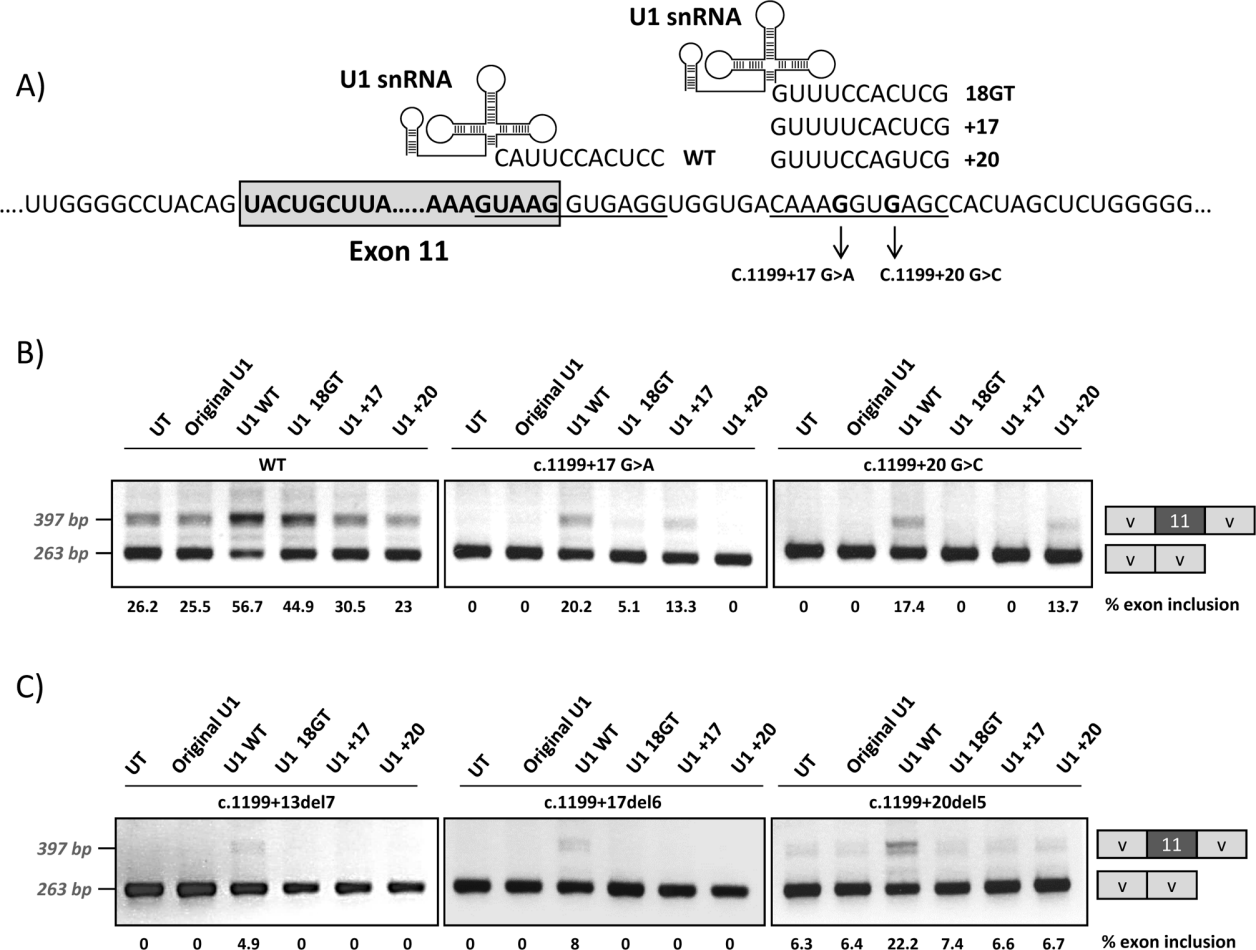
Co-transfection of wild-type and mutant minigenes with adapted U1snRNA constructs. Different modified U1 snRNA constructs were generated hybridizing to the 5’ splice site of *PAH* exon 11 (U1 WT), to the intronic cryptic splice site (U1 18GT), or to the intronic cryptic splice site with the mutations +17 (U1 +17) or +20 (U1+20), as shown in the upper panel (A). Panel B shows the results of co-transfecting the different U1 constructs in the wild type (wt) and mutant pSPL3 minigenes and panel C the results obtained with the minigenes carrying the intronic deletions c.1199+13del7, c.1199+17del6 and c.1199+20del5. On the right of the gel is the schematic drawing showing the identity of the bands. In panel B and C the estimated percentage of exon inclusion is shown below each lane.

We next generated different adapted U1 snRNAs to investigate whether we could correct the exon skipping defect of the +17 and +20 variants by forcing U1 binding to the cryptic splice site. This would further confirm that the pathogenic mechanism underlying the two mutations could be ascribed to deficient U1 binding to this region. The adapted U1 snRNAs exhibited perfect complementarity to the natural exon 11 5’ splice site (U1 WT), to the cryptic splice site at +18 (U1 18GT) or to the cryptic splice site at +18 with mutations +17 or +20 (U1 +17 and U1 +20) (Fig 7A). Fig 7B shows the results of the co-transfection experiments performed with the pSPL3 minigenes, where we observe complete exon skipping for the mutant minigenes (see Fig 1), thus facilitating the detection of even slight increases in exon 11 inclusion. Similar results were obtained with the pcDNA3 minigenes.

Adapted U1 fully complementary to the natural 5’ splice site (U1 WT) favoured exon inclusion for the wild type and mutant minigenes, as expected from previous studies in different genes, where this approach has been successfully used to correct splicing defects [14, 15]. Some positive effect was also observed for the wild type minigene when we co-transfected with the U1 18GT construct perfectly matching the cryptic splice site. For each mutant minigene, co-transfection of the corresponding adapted U1 (U1 +17 or U1 +20) resulted in increased exon inclusion (Fig 7B). The PCR bands corresponding to exon inclusion observed after cotransfecting the mutant minigenes with U1 WT, U1 +17 or U1 +20 were cloned and sequenced and in all colonies (30 for each), splicing occurred at the natural 5’ splice site. The fact that we observed that the U1 +20 exclusively corrects splicing from the +20 mutant minigene and that the U1 +17 exclusively corrects splicing from the +17 mutant minigene supports the notion that U1 binding at the +15_+24 site stimulates use of the normal 5’ splice site.

We also co-transfected the adapted U1 constructs along with the minigenes with the deletions described in Fig 2: c.1199+13del7, c.1199+17del6 and c.1199+20del5, to confirm that U1 +17 and U1 +20 are indeed binding to the intronic cryptic splice site and not elsewhere. As shown in Fig 7C only with U1 WT binding to the natural 5’ splice site exon inclusion increases.

## Discussion

Confirmation of the pathogenic nature of newly identified variants is mandatory in genetic diagnosis. For splicing defects, transcript analysis using patients’ cells or minigene-based assays provides information on the pathogenicity of the variant, prediction of disease severity and elucidation of the splicing mechanism. This provides insight on regulatory elements that define an exon and which are targets for designing specific therapeutic approaches [3]. In this work we have elucidated the pathogenic nature of two intronic point mutations in the *PAH* gene which are located outside the consensus 5’ splice site and intriguingly, caused skipping of the preceding exon 11. Aberrant exon skipping is a common splicing defect, resulting from different exonic or intronic mutations that ultimately disrupt the network of interactions that define an exon in a specific gene context. During spliceosome assembly, different positive and negative splicing factors are recruited to splice sites and adjacent regions through dynamic associations and their concerted action determines the final splicing output. In this work, we have identified a novel region in *PAH* intron 11 functioning as an ISE for the preceding exon 11 which has an intrinsically weak 3’ splice donor site. Deleting the intronic region or increasing the distance to the natural 5’ splice site causes exon skipping (Fig 2 and Fig 6). Of note, this ISE harbours a binding site for U1 snRNA and RNA affinity studies demonstrated that U1 70-K binds to this region in the wild-type context. This binding is lost in exon skipping mutants c.1199+17G>A and c.1199+20G>C which decrease the complementarity to U1 snRNA (Fig 4).

Further evidence of the role of intronic U1 binding for the correct recognition of the natural 5’ splice site of *PAH* exon 11 is provided by the results obtained after co-transfecting adapted U1 snRNA in mutant minigenes. Only the perfectly adapted U1 snRNA complementary to the c.1199+17G>A or c.1199+20G>C mutations favour exon inclusion in each case (Fig 7). In addition, abolishing the intronic U1 binding site in wild type minigenes increases residual exon skipping (Fig 5). On the contrary, increasing the complementarity in the intronic region to U1 snRNA results in complete exon inclusion (Fig 5).

Our results are in accordance to previous studies. Hwang and Cohen demonstrated that binding sites for U1 within exons or introns can act as splicing enhancers, compensating for substandard 3’ splice sites [26]. U1 may bind to different sites and contribute to exon definition by acting as coach for U6 binding nearby, thus directing the 5’ splice site choice which is determined by U6 [27]. In addition, using high-throughput RNA sequencing after RNA antisense purification techniques confirmed that U1 binds to 5’ splice site motifs throughout introns [28]. Eperon et al. found that U1 can bind simultaneously to alternative 5’ splice sites, as enhanced by SRSF1, and with simultaneous occupancy, the downstream 5’ splice site is preferentially used [29]. This occurred when the sites were > 40 nt apart and, in our study, we observe usage of the cryptic splice site with increasing distance to the natural site (Fig 6). Another study pointed to the role of hnRNPA1 with an antagonistic effect, interfering with U1 binding and, in that case, the splice site choice was shown to depend on the affinities of U1 for each site [30]. Consistent with this, our pull down studies indicated increased hnRNP A1 binding to the c.1199+20G>C mutant, thereby explaining the more severe effect of this mutant relative to the c.1199+17G>A despite its apparent less dramatic effect on the U1 motif strength. Indeed, the c.1199+20G>C change is predicted to increase the strength of hnRNP A1 binding motif (+1.36% according to HSF) while the c.1199+17G>A change abolishes two predicted binding sites albeit creating a new one (Table 1).

Pagani and co-workers identified U1 70K as mediator in the splicing rescue for exon skipping mutations of U1 binding at different sits in the intron [18]. These authors developed a therapeutic approach to correct exon skipping in different diseases based on modified U1 snRNA [17]. The second-generation modified U1 snRNAs, named Exon Specific U1snRNAs (ExSpeU1s), have engineered 5’ tails complementary to non-conserved intronic regions downstream of the 5’ donor splice site. Gene specific ExSpeU1s result in the assembly of a U1-like particle that rescues exon skipping mutations located in 5’ or 3’ splice sites or in exonic regulatory elements [17, 18]. In these studies, the splicing rescue activity is dependent on the U1-70K protein and on the loop IV structure of the U1 snRNA [18]. The U1-70K protein is known to interact through its RS-domain with RS-domain-containing splicing factors (SR proteins) bound in exons, favouring exon inclusion [31]. Remarkably, the reported ExSpeU1-mediated splicing correction appears not to require endogenous U1 snRNP, as assessed by U1 decoy experiments [18, 32]. This could indicate that the U1-like particles do not act by facilitating recruitment of the endogenous U1 to the upstream 5′ splice sites, but rather by promoting correct exon and intron definition, mainly through U1-70K and stem-loop IV elements, respectively [18]. In the *PAH* exon 11 sequence context, the operating mechanism may be similar; U1-70K protein binds to U1 snRNA bound at the downstream intronic site initiating the formation of the correct network of splicing factors over the exon. Adapted U1 snRNAs that bind to the mutant c.1199+17A or c.1199+20C intronic sites (Fig 7) compensate by recruiting U1-70K protein thereby reconstituting the missing interactions that define exon 11.

A recent study showed that the rescue of disease-causing splicing mutations by ExSpeU1 snRNA in coagulation factor IX (FIX) exon 5 is mediated by an SRSF2-dependent enhancement mechanism [33]. SRSF2 exhibits weak binding to wild type *PAH* intron 11 region, which is abolished with mutations c.1199+17G>A or c.1199+20G>C (Fig 4), arguing in favour of its involvement in correct exon definition. We also detected increased binding of splicing inhibitory hnRNPA1 protein to the +20G>C mutant sequence (Fig 4), which could also contribute to the exon skipping effect. We speculate that also a balance exists between inhibitory binding of hnRNP A1 to the motifs in the +14 –+32 region (Fig 4) and binding of U1 at the cryptic splice site. In the normal situation, one of the roles of U1 binding at the cryptic site could thus be to avoid inhibitory binding of hnRNP A1 to the motifs in the +14 –+32 region. Thus, several regulatory mechanisms may be acting in concert for correct exon definition and mediating in the pathogenic effect of the described variants.

In summary, this work provides additional evidence to understand the mechanisms underlying correct exon definition through the involvement of splicing regulatory elements located outside the splice sites. Importantly, it also sheds light on the mechanism underlying the correcting effect of the ExSpeU1s, by demonstrating that in certain contexts, U1 snRNP can act as a splicing stimulator when bound to an intronic region flanking the natural 5’ splice site.

## Materials and methods

### Cell culture and conditions

Human hepatoma cell lines, Hep3B and HepG2, were grown in Minimum Essential Medium (MEM, Sigma Aldrich) supplemented with 5% fetal bovine serum (FBS), 1% glutamine and 0.1% antibiotic mix (penicillin/streptomycin) under standard cell culture conditions (37°C, 95% relative humidity, 5% CO_2_).

### Minigenes construction

For evaluation of in vitro splicing two different minigenes constructs were used. In the first construct (pSPL3 minigene), a fragment of human *PAH* including intron 10 reduced to 92 bp (normal length is 556 bp), exon 11 and intron 11 reduced to 100 bp (full length is 3130), was amplified using primers located in intron 10 (5’-TGAGAGAAGGGGCACAAATG-3’) and in intron 11 (5’-GTAGACATTGGAGTCCACTCT-3’). Gene fragment and flanking region was cloned into the pGEMT vector (Promega). The insert was excised with EcoRI and subsequently cloned into pSPL3. The second construct (pcDNA3.1 minigene) includes exon 10, full intron 10, exon 11, 1958 bp of intron 11, and exon 12 cloned in pcDNA3.1+ [22].

Variant minigenes containing mutations c.1199+17G>A and c.1199+20G>C were generated by site-directed mutagenesis with QuikChange Lightning Kit (Agilent Technologies, Santa Clara, CA) using primers 5’-GTGAGGTGGTGACAAAAGTGAGCCACTAGCTC-3’ and 5’- GTGAGGTGGTGACAAAGGTCAGCCACTAGCTC-3’, respectively, and their reverse complement. For deletions, we used primers c.1199+13_1199+19del (5’-AAGGTGAGGTGGTGAGAGCCACTAGCTCTG-3’), c.1199+17_1199+22del (5’-AAGTAAGGTGAGGTGGTGACAAACCACTAGCTCTG-3’) and c.1199+20_1199+24del (5’-AGGTGAGGTGGTGACAAAGGTACTAGCTCTGGG-3’), and their reverse complement. To optimize the 5’ splice site the c.1199+3G>A_+6G>T mutations were introduced in wild-type minigene using primer 5’- GAGTTTTAATGATGCCAAGGAGAAAGTAAGGTAAGTTGGTGAC-3’ and its reverse complement. We also introduced changes at the cryptic intronic splice site: c.1199+18G>C and c.1199+15 A>C/+20G>A using primers 5’-GAGGTGGTGACAAAGCTGAGCCACTAGCTCT-3’ and 5’-GAAAGTAAGGTGAGGTGGTGACACAGGTAAGCCACTAGCTC-3’ respectively, and their reverse complement. Spacers were introduced by site-directed mutagenesis.

### U1snRNA constructs

The parental U1 snRNA clone was pG3U1 (original U1) [34], a derivative of pHU1 [35]. We created the variants U1 WT, U1 18GT, U1 MUT+17, and U1 MUT+20 by replacing the sequence between the *Bcl*I and *Bgl*II sites with mutant oligonucleotides with perfect complementarity to exon 11 5’ splice site (U1 WT), to the intronic cryptic splice site (U1 18GT), and to the intronic cryptic site with mutations +17 (U1 MUT+17) or +20 (U1 MUT+20).

### Transient transfections and splicing analysis

For minigene assays, Hep3B cells were seeded in six-well plates at a density of 4x10^5^ in 2 ml 5% MEM and grown overnight. Cells were transfected with a total DNA amount of 2 μg per well using JetPei DNA Transfection Reagent (Polyplus, NewYork). For U1 snRNA overexpression experiments cells were transfected with 1 μg of wild type or mutant minigenes and co-transfected with 1 μg of U1 snRNA variants. Cells were harvested by trypsinization after 48 h. Total RNA was isolated using Trizol Reagent (ThermoFisher) and phenol-chloroform extraction. cDNA synthesis was performed using NZY First-Strand cDNA Synthesis Kit (NZYtech). Splicing analysis was carried out by PCR amplification with FastStart Taq Polymerase (Roche) using specific primers to exclude detection of endogenous *PAH* gene expression: SD6 (5’-TCTGAGTCACCTGGACAACC-3’) and SA2 (5’-ATCTCAGTGGTATTTGTGAGC-3’) for pSPL3 minigene, and *PAH* 10-11-12 S (5’-GGTAACGGAGCCAACATGGTTTACTG-3’) and *PAH* 10-11-12 AS (5’- AGACTCGAGGGTAGTCTATTATCTGTT-3’) for pcDNA3.1 minigene. The end-point PCR amplification products were analyzed by 2% agarose gel electrophoresis and/or by capillary gel electrophoresis using the Fragment AnalyzerTM (Advanced Analytical), and their identity was confirmed by Sanger sequencing. The experiments were performed at least two times. The relative quantity of the bands corresponding to exon inclusion/exon skipping was estimated by laser densitometry using ImageLab software and reported as percent exon skipping (relative to the sum of both bands in each lane).

Splicing analysis of endogenous *PAH* transcripts was performed in Hep3B and HepG2 cell lines and in an anonymized human liver sample obtained from the diagnostic laboratory CEDEM in Madrid. For cycloheximide treatment, 40 μg/ml of cycloheximide was added to the culture media 6 hours prior to harvest. RNA extraction was performed as described above and primers hybridizing to exon 10 (5’-ACTGTGGAGTTTGGGCTCTG-3’) and exon 12 (5’-ACTGAGAAGGGCCGAGGTAT-3’) were used for amplification.

### RNA oligonucleotide affinity purification

Affinity purification of RNA binding proteins was performed with 3’-biotin coupled RNA oligonucleotides (LGC Biosearch Technologies, Denmark) as previously described [22]. Sequences of the RNA oligonucleotides were: *PAH*-wt (5’-UGACAAAGGUGAGCCACUAG-3’), *PAH*-mut+17 (5’-UGACAAA**A**GUGAGCCACUAG-3’) and *PAH*-mut+20 (5’-UGACAAAGGU**C**AGCCACUAG-3’) corresponding to position c.1199+10_1199+29 of *PAH* mRNA. For each purification 100 pmol of RNA oligonucleotide were coupled to 100 μl of streptavidin-coupled magnetic beads (Invitrogen) and incubated with Hela nuclear extract (Cilbiotech S.A., Belgium). Eluted proteins were analyzed by western blotting using antibodies against SRSF1 (32–4500 from Zymed Laboratories (Invitrogen)), SRSF2 (04–1550 from Millipore), SRSF5 (H6430-M03A from Abnova), SR proteins (33–9300 from Invitrogen), SRSF7, U1snRNP70, Tra-2β, hnRNPA2/B1, hnRNPI, hnRNPL, hnRNPH, hnRNPE2 (sc-10244, sc-9571, sc-33318, sc-53531, sc-16547, sc-32317,sc-28380,sc-10042, and sc-101136 from Santa Cruz Biotechnology) and hnRNPA1(R9778 from Sigma Aldrich). The gels were stained with Coomassie solution (0.1% Coomassie Brilliant R-250 Blue, 50% methanol v/v, 10% glacial acetic acid) and destained in a 40% methanol and 10% glacial acetic acid solution. The pictures were acquired on a Gel Doc XR+ System (from Bio-Rad). Quantification of the western blots bands was performed on duplicates by calculating the levels of grey using ImageJ software (https://imagej.nih.gov/ij).

### In silico splicing prediction

The effect of the variants on the splice site strengths and the presence of putative splicing regulatory elements were predcited using the Human Splicig Finder (HSF) program (http://www.umd.be/HSF3/) [36], MaxEntScan software (http://genes.mit.edu/burgelab/maxent/Xmaxentscan_scoreseq.html) [37], the BerkeleyDrosophila Genome Project (BDGP) splice prediction tool (http://www.fruitfly.org/seq_tools/splice.html) [38] and ESEFinder 3.0 software (http://rulai.cshl.edu) [39].
